# Good Equivalence Between Electronic and Paper Versions of the Measure Yourself Medical Outcome Profile 2 and the Measure Yourself Concerns and Wellbeing: A Mixed Methods Study

**DOI:** 10.7759/cureus.77825

**Published:** 2025-01-22

**Authors:** Maria Lund-Tonnesen, Birthe T Oggesen, Susanne V Lauridsen, Siv Fonnes, Jacob Rosenberg

**Affiliations:** 1 Surgery, Center for Perioperative Optimization, Herlev Hospital, Herlev, DNK; 2 Surgery, Copenhagen Sequelae Center CARE, Herlev Hospital, Herlev, DNK; 3 Urology, Herlev Hospital, Herlev, DNK; 4 Rheumatology, The Parker Institute, Bispebjerg and Frederiksberg Hospital, Frederiksberg, DNK; 5 Health and Medical Science, University of Copenhagen, Copenhagen, DNK

**Keywords:** equivalence study, mycaw, mymop2, patient-reported outcomes, reliability and agreement

## Abstract

Aim/background

Measure Yourself Medical Outcome Profile 2 (MYMOP2) and Measure Yourself Concerns and Wellbeing (MyCaW) are two questionnaires that allow patients to define symptoms and concerns that are important to them. As the equivalence between the electronic and paper versions has not been assessed, we aimed to determine the measurement equivalence of electronic and paper versions of MYMOP2 and MYCaW among a group of adult patients treated for colorectal or anal cancer. We also aimed to investigate the patients’ experiences of filling out the questionnaires.

Methods

A mixed methods approach was chosen to investigate measurement equivalence and to ensure a thorough investigation of the patients’ experiences. The intra-rater reliability for ordinal data was assessed using the Intraclass Correlation Coefficient (ICC) and median difference. Agreement in descriptive data was assessed by two researchers independently. To capture the patients’ experiences, we conducted semi-structured interviews over the phone. Interviews were transcribed verbatim and analyzed with content analysis. Results were presented as themes.

Results

A total of 30 patients were enrolled consecutively from a sequelae center treating patients after colorectal or anal cancer. Of these, 19 patients were also interviewed. The patients filled out MYMOP2 and MYCaW electronically and on paper. The intra-rater reliability for ordinal data was either excellent (n=11) or good (n=3) with ICCs ranging from 0.85 to 1. The median difference between ordinal data from electronic and paper versions was 0 (range -4 to 4). The agreement between the electronic and paper versions in the descriptive data ranged from 70% to 97%, with three-quarters being over 80%. Three themes emerged from the interviews: personal preferences, the importance of setup, and the availability of the questionnaires. Most patients preferred electronic versions as the advantages of electronic questionnaires outweighed the paper versions.

Conclusion

We found good intra-rater reliability and agreement in MYMOP2 and MYCaW questionnaires, demonstrating a high measurement equivalence. The interviews provided perspectives on the user experience and showed a preference for the electronic questionnaires as they had more advantages such as availability.

## Introduction

Patient-reported outcomes (PROs) reflect the patients’ perspectives and are important in assessing symptoms and other aspects of treatment [1]. Many PRO questionnaires only have one focus, for example, symptoms, health-related quality of life, or patient experience of care [2]. Measure Yourself Medical Outcome Profile 2 (MYMOP2) [3] and Measure Yourself Concerns and Wellbeing (MYCaW) [4] questionnaires let the patients define their most bothering symptoms or concerns and assess their feeling of well-being, thus providing a holistic approach to patient involvement. MYMOP2 [3] is an updated version of the original MYMOP from 1996 [5]. It asks patients to identify bothersome symptoms, describe activities that are made difficult by these symptoms, and indicate how long they have been experiencing them. Additionally, patients are asked to rate their overall well-being. MYMOP2 [3] also includes a question about the use of medication for these symptoms and whether it is important to reduce or avoid taking the medication. MYCaW [4] asks the patients to define concerns or problems and rate their general well-being. Both questionnaires were initially developed in paper format; however, with the increasing digitalization of healthcare systems, including the Danish healthcare system [6,7], and the growing digital interaction between patients and healthcare providers, there is now a demand for electronic questionnaires. Furthermore, MYMOP2 is increasingly used in research [8], and the use of electronic PROs reduces the risk of typing errors when converting written text to electronic data that can be analyzed and interpreted [9]. By assessing the measurement equivalence of PRO questionnaires’ electronic versus paper versions, we ensure comparable results that support using the questionnaires in multiple ways [10]. This has been demonstrated in other studies [11-13], but measurement equivalence has not been tested in MYMOP2 or MYCaW.

We aimed to determine the measurement equivalence between electronic and paper versions of MYMOP2 and MYCaW questionnaires among a group of adult patients treated for colorectal or anal cancer and suffering from sequelae. We also aimed to investigate the patient experiences of filling out the questionnaires.

## Materials and methods

Study design

A mixed methods approach was chosen as we wanted to investigate both the measurement equivalence and the patients’ experiences of filling out the questionnaires. This study was reported according to the Guidelines for Reporting Reliability and Agreement Studies (GRRAS) [14] and COnsolidated criteria for REporting Qualitative research (COREQ) [15]. To test intra-rater reliability and agreement, each patient served as his or her own control and, therefore, had to fill out both the electronic and the paper versions. When doing so, it is advised to use a crossover design [16]. We, therefore, chose a two-sequence crossover design for the baseline questionnaires, where every other patient filled out the electronic versions first and every other filled out the paper versions first, alternately. The crossover design did not apply to the follow-up questionnaires, as they were filled out by patients in their homes without supervision. The experiences of using the questionnaires were explored via semi-structured interviews conducted over the phone one week after the baseline questionnaires were filled out. The study was approved by the Danish Data Protection Agency (p-2023-14768). Ethical approval is not required nor possible according to Danish legislation for neither questionnaires nor interview studies [17]. Participants were informed verbally and in writing, and they signed an informed consent form when entering the study. Data have been treated confidentially, and all participants were anonymized.

Setting and participants

The study took place in a single-center setting at Copenhagen Sequelae Center CARE (Colon-, Anal-, and REctal cancer) at Herlev Hospital in Denmark [18] and from the patients’ homes. The patients participated in the study before or after attending their appointments. The data collection period was November 29, 2023, to March 13, 2024. Patients were enrolled by consecutive sampling. The inclusion criteria were patients treated for colorectal or anal cancer who were able to read and speak Danish and were 18 years of age or older. The exclusion criteria were not having any electronic devices at home, not speaking Danish, or being unreachable to schedule an appointment. As we aimed to determine the measurement equivalence rather than a difference based on a fixed hypothesis, we did not calculate a sample size. Instead, the robustness of the study's findings was analyzed, including 95% CIs, and the sample size of 30 participants was deemed sufficient, supplemented by qualitative data from the semi-structured interviews. The number of interviewed patients was determined based on the principle of data saturation [19], which was discussed and agreed upon by the researchers.

MYMOP2 and MYCaW questionnaires

As MYMOP2 and MYCaW were translated into Danish in 2022 [20], these validated versions were used in the study and both consisted of a baseline and a follow-up questionnaire.

In the MYMOP2 [3] baseline questionnaire, patients are asked to define one to two bothering symptoms and rate these on a 0- to 6-point Likert scale. In addition, patients are asked to write an activity made difficult or impossible because of the symptom(s) and rate this on a 0- to 6-point Likert scale. They rate their general well-being over the past week on a 0- to 6-point Likert scale and select one of five options regarding how long symptom one has been bothering them. In the Danish version, the questions about medication differ; patients are instead asked whether they have treated their sequelae, and if so, how, as well as what consequences stopping the treatment might have [20]. In the MYMOP2 follow-up questionnaire, patients re-score their earlier indicated two symptoms and the activity as well as rate their general well-being again on a 0- to 6-point Likert scale. A third symptom can be added and scored. In the follow-up questionnaire, instead of sequelae, the patients are asked if anything else is affecting their problem, if they take medicine, and if they do, what medicine and how much [20].

In the Danish translation of MYCaW [20], patients are asked to describe one to two concerns or problems and rate these on a 0- to 6-point Likert scale. They also rate their well-being on a 0- to 6-point Likert scale. In the Danish MYCaW follow-up questionnaire, the patients re-score their earlier concerns or problems and re-score their well-being.

Data collection

In the outpatient sequelae center, patients filled out MYMOP2 and MYCaW baseline questionnaires both electronically and on paper in a crossover setup where they started alternately with the electronic or paper versions. Neither patients nor researchers were blinded. The electronic versions could be assessed on a computer, a tablet, or on the patients’ smartphones in the sequelae center. Patients were encouraged to use the same device as they would at home. The electronic questionnaires and patient characteristics were collected and managed via Research Electronic Data Capture (REDCap) [21,22] hosted by the Capital Region of Denmark. Patients filled out the baseline questionnaires independently with researcher MLT present to aid with possible questions; one patient had his wife present as well. Patients were given the follow-up paper questionnaires and a return envelope in the sequelae center to return the following week. The electronic follow-up questionnaires were sent automatically five days after the baselines were filled out. Some patients returned paper questionnaires by letter but most sent a picture of the paper questionnaire via e-mail. If the patients did not return the electronic and/or paper follow-up questionnaires within a few days after the electronic version was sent, they were contacted by phone or e-mail and reminded.

Qualitative methods

To gain knowledge about the patients’ experiences of filling out MYMOP2 and MYCaW electronically and on paper we used an inductive approach [23]. A semi-structured interview guide [24] was developed within the author group, with a focus on an open and curious approach. The main focus was the patients’ experiences with completing the electronic and paper questionnaires and how they differed. An English version of the interview guide can be seen in Table 4 of the Appendices. Interviews were conducted in Danish by the first author, a registered nurse (RN) and PhD student affiliated with the sequelae center, but with no involvement in direct patient care. Interviews were conducted one week after the baseline data collection via phone, and they were audio recorded and transcribed verbatim using artificial intelligence [25]. Phone interviews were chosen because of the availability, as the interviews had to take place when the patients still had the questionnaires in fresh memory. The interviews had a median duration of 10 minutes (range: six to 20). After the first five interviews, MLT and SVL, both with previous experience in qualitative methods, assessed the transcriptions and evaluated the interview guide with minor adaptions. There were no follow-up interviews, and transcripts were not sent to patients for comments. The qualitative transcribed data from the semi-structured interviews were coded by MLT and SVL using the principles of content analysis [26]. When using an inductive approach, categories are derived from the data, moving from the specific to the general [23]. The first step of the analysis involved reading all the transcripts to gain an overall sense of the data. The second step was identifying meaning units in the form of quotes, which were then condensed and assigned a code. Finally, the codes were organized into themes [26]. An example of a coding tree can be seen in Table 1.

**Table 1:** Coding tree example.

Quote, participant ID	Condensed meaning	Code	Theme
“(…) the paper version takes longer to get out to one,” ID 3	Paper versions take longer to receive	Receive	Availability
“(…) could send immediately [electronic versions],” ID 5	Easy returning electronic versions	Return	
“Then you just sit down for 5 minutes, fill it in, send it off, and don’t think about it anymore,” ID 11	You can fill out and return the electronic versions easy and fast	Return	
"(…) you can also do it on the go, you can do it in the car or the train or the garden,” ID 18	You can fill out the electronic versions anywhere	Flexibility	
“It becomes a bit more difficult if you have to send something [paper] in,” ID 17	Paper versions are more difficult to return	Return	

Statistical analyses

All quantitative data were analyzed using SPSS (version 29.0, IBM Corp., United States). Ordinal data from the Likert scale questions were analyzed for intra-rater reliability using the Intraclass Correlation Coefficient (ICC), calculated with a two-way mixed-effects model and absolute agreement, as recommended for assessing intra-rater reliability [27]. Ordinal data are presented as ICC scores and 95% CIs. If data were missing in the electronic and/or paper questionnaires, these were excluded from the ICC calculations. Therefore, the number of included patients in each ICC calculation is presented as numbers and percentages. Since the Likert scale questions represented a well-defined continuum and assumed that the intervals between points were equal, we treated the ordinal data as continuous, allowing for the calculation of the median difference, which was chosen because the data were not normally distributed. This was tested using Q-Q plots and histograms. The number of included patients contributing data for median differences is also presented as numbers and percentages, as some patients rated 0 on one questionnaire and provided no response on the other. The nominal data from the five options of "How long did you have symptom 1?" are presented as numbers and percentages in terms of agreement. Descriptive data, such as symptoms, were rated independently by researchers MLT and SF for conformity and wording in three categories: "same answer" if the answers were identical, "same meaning" if different wording was used but the meaning was the same, and "not the same answer" if different wording and different meanings were used. Disagreements were resolved through discussion until a consensus was reached. The categories "same answer" and "same meaning" are pooled, and the proportion of agreement and disagreement is presented as numbers and percentages. If a patient left an answer blank in both the electronic and paper questionnaires, these were interpreted as "same answer."

## Results

**Figure 1:**
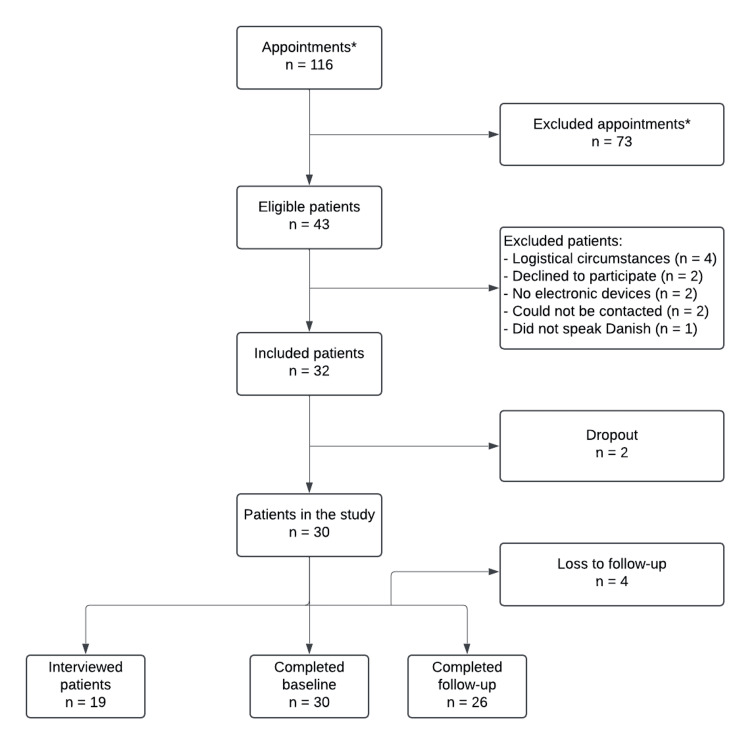
Flow diagram showing the patient sampling. We prioritized including patients who had already attended a physical appointment at the sequelae center. If there were many telephone appointments, patients were contacted and asked if they would prefer to come to the sequelae center instead. *During the sampling period, some patients had multiple appointments, so the 116 appointments do not correspond to 116 individual patients.

A total of 160 appointments were screened for eligibility, and from these, 30 patients were enrolled and included. Of these, 19 patients participated in both the questionnaire data collection and the interviews (see Figure 1). The response rate was 100% for both the electronic and paper baseline questionnaires, 100% for the electronic follow-up questionnaires, and 87% for the paper follow-up questionnaires, as four patients did not return the paper versions. These four follow-up questionnaires (both electronic and paper versions) were excluded from the data analysis. The returned follow-up questionnaires were completed later than the electronic versions, with a median delay of 0 days, ranging from 0 to 32 days (Table 5 of the Appendices).

Of the 30 included patients, 20 (66%) were female and the median age was 63 years (range: 34-77). Three of the patients were dyslexic and two had visual impairment, but they all understood the questions and were able to complete the questionnaires. The group of interviewed patients did not differ in characteristics, consisting of 12 females (63%) and a median age of 66 years (range: 34 to 77).

Measurement equivalence

Ordinal data from all the 15 questions based on a Likert scale across questionnaires are presented in Table 2. The intra-rater reliability measured with ICC was excellent (ICC >0.9) for most questions (n=12) and good (ICC: 0.75-0.90) for the remaining (n=3). This was also confirmed by a median difference on 0 (range: -4-4) across all 15 questions on the 0- to 6-point Likert scale (Table 2). The overall ICC scores were high, with 13 out of 15 questions having a 95% CI within the cut-off for either good or excellent inter-rater reliability. There were two questions with wider confidence intervals. One was the question on well-being in the MYMOP2 baseline questionnaire, where the 95% CI ranged from 0.62 to 0.93. Of the 30 patients, one had a difference of five between paper and electronic versions, 15 patients scored the same, 12 patients scored +1/-1, and two patients scored +2/-2. The second question was the nominal variable, "How long have you had symptom 1?" from the MYMOP2 baseline questionnaire. Patients could choose between five answers. All but one patient, 29 (97%), answered the same in the electronic and the paper versions.

**Table 2:** Equivalence of ordinale data from electronic and paper Likert scale items. Equivalence of ordinale data from electronic and paper Likert scale items on baseline and follow-up questionnaires for MYMOP2 [3] and MyCaW [4]. The Likert scale was a 0- to 6-point scale, and the median is summarized; the range for all questions was 0-6. ^A^The ICC describes the extent of agreement and similarity between measurements [27]. The definition of ICC values is as follows: <0.5 indicates poor reliability, 0.5-0.75 indicates moderate reliability, 0.75-0.90 indicates good reliability, and >0.90 indicates excellent reliability [27]. ^B,C^As four patients were lost to follow-up (n=26). ^D^The number of patients included in the ICC calculation depended on the missing data in one or both questionnaires. MYMOP2, Measure Yourself Medical Outcome Profile 2; MyCaW, Measure Yourself Concerns and Wellbeing; ICC, intraclass correlation coefficient

Questionnaire: item			Electronic		Paper		Median difference (range)
	n (%)^D^	ICC^A^ (95% CI)	n (%)	Median	n (%)	Median	
MYMOP2 baseline:							
Symptom 1	30 (100)	0.98 (0.95–0.99)	30 (100)	5	30 (100)	4	0 (-1–1)
Symptom 2	26 (87)	0.92 (0.81–0.96)	26 (87)	4	26 (87)	4	0 (-3–1)
Activity	27 (90)	0.95 (0.89–0.98)	27 (90)	4	28 (93)	5	0 (-1–1)
Wellbeing	30 (100)	0.85 (0.62–0.93)	30 (100)	3	30 (100)	4	0 (-4–1)
MYMOP2 follow-up^B^:							
Symptom 1	26 (100)	0.94 (0.86–0.97)	26 (100)	4	26 (100)	4	0 (-2–2)
Symptom 2	23 (86)	0.97 (0.92–0.99)	23 (88)	3	23 (88)	3	0 (-2–2)
Symptom 3	2 (8)	1.00	4 (15)	4	3 (12)	4	0 (0–0)
Activity	24 (92)	0.90 (0.77–0.96)	24(92)	3	25 (96)	4	0 (-3–1)
Wellbeing	26 (100)	0.96 (0.92–0.98)	26 (100)	3	26 (100)	3	0 (-1–1)
MyCaW baseline:							
Concern 1	30 (100)	0.95 (0.89–0.98)	30 (100)	5	30 (100)	5	0 (-1–2)
Concern 2	21 (70)	0.89 (0.74–0.96)	23 (77)	4	22 (73)	5	0 (-3–1)
Wellbeing	28 (93)	0.92 (0.83–0.96)	28 (93)	3	30 (100)	3	0 (-1–4)
MyCaW follow-up^C^:							
Concern 1	26 (100)	0.96 (0.92–0.98)	26 (100)	4	26 (100)	4	0 (-2–1)
Concern 2	18 (69)	0.94 (0.85–0.98)	18 (69)	4	21 (81)	3	0 (-1–2)
Wellbeing	25 (96)	0.96 (0.91–0.98]	26 (100)	3	25 (96)	3	0 (-1–2)

Agreement in the descriptive data between the electronic and paper versions, based on answers provided by the patients, is shown in Table 3. Agreement was assessed in 12 questions and ranged from 70% to 97%, with three-quarters of the questions showing agreement above 80%, indicating a high degree of agreement between the electronic and paper versions of the questionnaires.

**Table 3:** Measurement of agreement in descriptive data. Measurement of agreement in descriptive data between electronic and paper versions of the MYMOP2 [3] and MYCaW [4] questionnaires. Baseline questionnaires (n=30). Data are presented as numbers (%). ^A,C^Follow-up questionnaires (n=26), as four participants were excluded as they did not return the paper questionnaires. ^B^In the questionnaire it is formulated as: “The treatment you are receiving may not be the only thing affecting your problem. If there is anything else that you think is important, such as changes you have made yourself, or other things happening in your life, please write it here (write overleaf if you need more space):________” MYMOP2, Measure Yourself Medical Outcome Profile 2; MyCaW, Measure Yourself Concerns and Wellbeing

Questionnaire: item	Same answer	Same meaning	Not the same answer
MYMOP2 baseline:			
Symptom 1	15 (50)	14 (47)	1 (3)
Symptom 2	14 (47)	13 (43)	3 (10)
Activity	14 (47)	13 (43)	3 (10)
Have you treated your sequelae?	14 (47)	9 (30)	7 (23)
What happens if you end the treatment?	15 (50)	10 (33)	5 (17)
MYMOP2 follow-up^A^:			
Symptom 3	21 (81)	1 (4)	4 (15)
Other important things^B^	22 (85)	1 (4)	3 (11)
Are you taking medication for this problem?	21 (81)	3 (11)	2 (8)
MYCaW baseline:			
Concern or problem 1	8 (27)	16 (53)	6 (20)
Concern or problem 2	14 (47)	7 (23)	9 (30)
MYCaW follow-up^C^:			
Other things affecting your health?	17 (65)	3 (12)	6 (23)
What has been most important for you?	8 (31)	13 (50)	5 (19)

Content analysis

**Figure 2:**
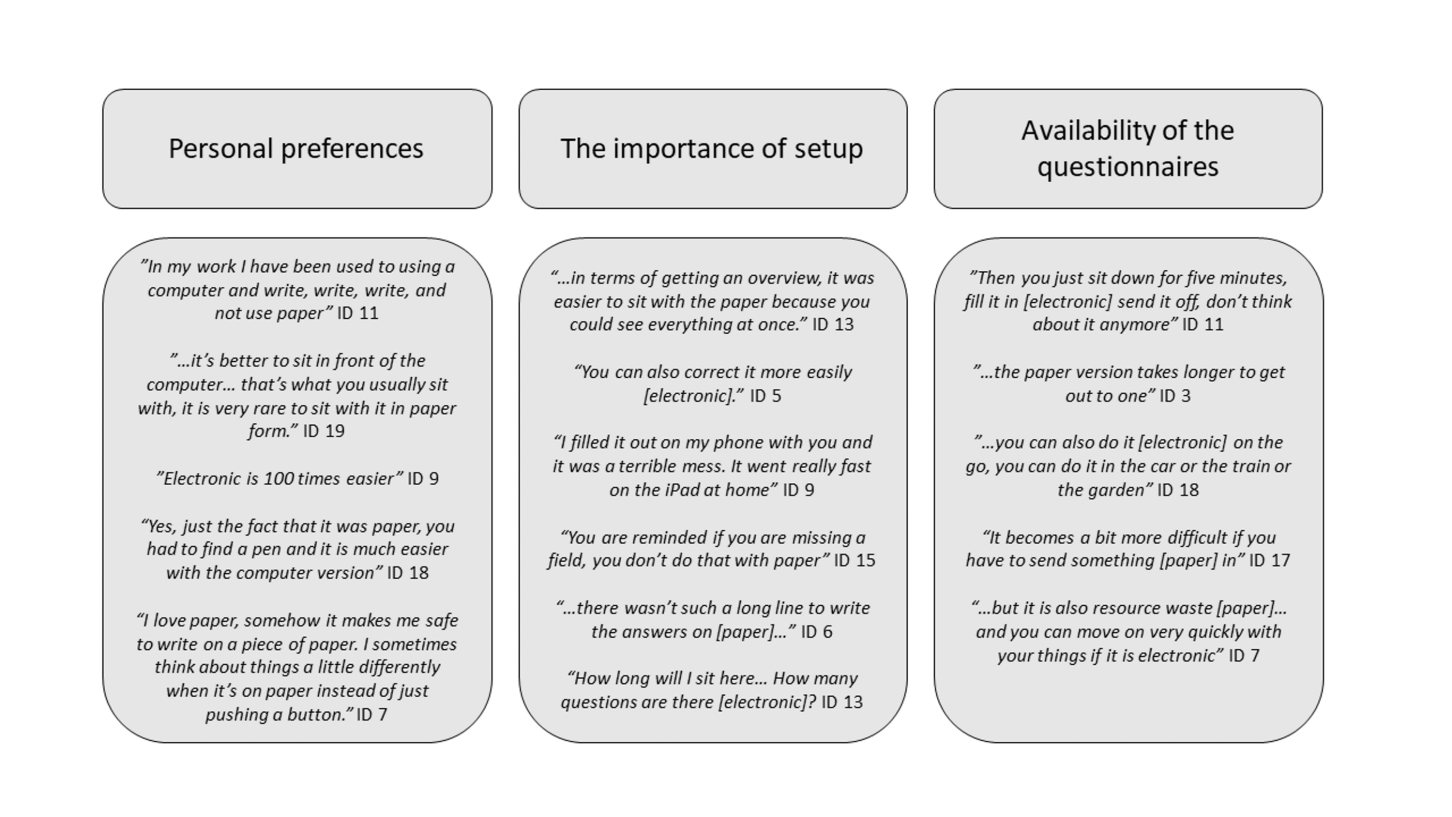
The results of the thematic analysis are divided into three themes and their quotes.

The content analysis of the qualitative data identified three themes: personal preferences, the importance of setup, and the availability of the questionnaires. The themes are presented below and exemplified with quotes in Figure 2.

Personal Preferences

The patients’ preferences were influenced by habits, personal likings, and what felt easy regarding preference for the electronic or paper versions. Some patients explained that they preferred the electronic versions as they were frequently using electronic devices. It was also described as being easier and quicker. Some patients preferred paper, explaining this based on paper feeling nice, safe, cozy, being old fashioned, and that sitting with paper gave better reflections, see examples in Figure 2.

The Importance of Setup

Experiences with the setup were described in terms of overview and layout. Some patients described a lack of overview in the electronic versions in terms of how long they were. This was at the same time emphasized as an advantage in the paper questionnaires. In terms of the layout, the electronic versions offered plenty of room for writing free text as opposed to the paper versions. The electronic questionnaires also alerted if a question was missed. This was for some articulated as an advantage and for some as being annoying because it did not point out which field was missing. Some participants found it troublesome to write in hand and found their own handwriting hard to read. Several patients had problems with the electronic versions when opened on a smartphone as the design was web-based, e.g., the smartphone layout made it hard to read the question above the Likert scale, see Figure 2.

Availability of the Questionnaires

Many patients emphasized that the availability and ease of returning the electronic version was a major advantage, as they could submit it immediately after completing it. Returning the paper version was seen as slow, troublesome, and not reliable. If the questionnaires were to be received by mail, similar problems were pointed out. The flexibility to fill out the electronic versions on the go was also highlighted, which is exemplified in Figure 2. Furthermore, some articulated paper versions as a waste of resources.

When the patients were asked which version of the questionnaires they preferred, electronic or paper, 15 of the 19 interviewed patients preferred the electronic version, two preferred the paper version, and two did not care which version was used.

## Discussion

There was "excellent" or "good" intra-rater reliability for the majority of the ordinal data between the electronic and paper versions of both the MYMOP2 and MYCaW questionnaires. Additionally, the agreement of the descriptive data across the electronic and paper versions showed a high degree of consistency. The qualitative data indicated that patients appreciated the availability of the electronic versions, as it allowed them to complete the questionnaires at their convenience and submit them immediately. This was highlighted as a significant advantage compared to receiving a paper version and returning it by mail.

The high degree of measurement equivalence found between the electronic and paper versions in our study is consistent with other studies testing measurement equivalence between electronic and paper questionnaires [11-13]. This confirms that questionnaires can be transitioned to other administration modes with a high degree of inter-rater reliability and agreement, as long as the design and setup closely resemble each other [16]. This measurement equivalence supports the possibility of distributing MYMOP2 and MYCaW electronically. Several advantages arise from this, including greater flexibility, which aligns with the growing use of telemedicine [28], offering enhanced convenience for patients, as expressed in the interviews. This is particularly relevant in healthcare settings using online or phone consultations, or those with short-duration consultations [18], ensuring efficient and effective collection of PROs. Another advantage is the ability for both patients and healthcare practitioners to prepare before the consultation. This could result in a more accurate and personalized consultation, and it may also be less time-consuming. However, this assumes that patients can be contacted digitally before the consultation, which requires a registry or another form of organized patient data management. Another potential challenge is digital health literacy, as some patients do not use electronic devices. Even though the questionnaires can still be filled out during the consultation, this may disadvantage these patients due to health inequalities [28]. When data are collected electronically, it is easier to compare and analyze for both improving everyday practice and research purposes. However, it requires a digital setup with equipment and a database, such as REDCap, to manage data securely, which some healthcare settings may not have.

The patients experienced several advantages with the electronic versions of the questionnaires, both in terms of convenience and availability. This provided important information that we would not have received otherwise, demonstrating, among other things, that receiving and returning questionnaires electronically was much easier and more manageable for patients than doing so via mail, even though the digital setup did not function optimally on smartphones. Most of the interviewed patients preferred the electronic versions, which is consistent with other studies asking patients about their preference between electronic and paper versions of PROs [29]. As telemedicine, such as phone and online consultations, becomes more widely used [6,7], electronic versions of questionnaires are essential for collecting PROs for both clinical practice and research purposes. This aligns with the general digital progress in society, which is reflected in patients' habits and preferences for using electronic devices. Since many of the consultations at the outpatient sequelae center are phone consultations [18], this study paves the way for using MYMOP2 and/or MYCaW in this setting.

The strength of this equivalence study lies in its mixed methods approach, which investigates both measurement equivalence and patients’ experiences to provide nuances and insights. By using this approach, the study highlights both the possibility and advantage of using electronic questionnaires. The study tested measurement equivalence in a homogeneous patient group with colorectal or anal cancer, focusing on sequelae after treatment. By testing equivalence in a homogeneous group with similar symptoms, the internal validity was strengthened. Ratings of the descriptive data were done independently by two authors to ensure objectivity and avoid misinterpretations. Regarding the qualitative interviews and analysis, both MLT and SVL had prior experience with qualitative methods. The study had a very high response rate, with 100% for the baseline questionnaires and an 87% response rate for the follow-up questionnaires, which is considered excellent for questionnaire data [30]. Finally, the two questionnaires used in this equivalence study are validated tools. The original MYMOP was validated against the SF36 health survey [5], and MYCaW was validated against FACIT-SPEx [4], with the Danish versions also having been validated [20].

This study also has some limitations. We did not perform a sample size calculation; however, most of the ICC 95% CIs were good or excellent, suggesting that a larger sample would likely have yielded similar results, only narrowing the 95% CIs. Although a crossover design was used, the order in which patients filled out the electronic and paper versions was not randomized but alternated. The lack of randomization could potentially introduce confounding variables that may influence the results. Patients completed the electronic and paper baseline questionnaires immediately after one another, without a wash-out period in between, as recommended in a crossover design [16,31]. The absence of a wash-out period could have affected patient responses, as the wash-out period is intended to minimize the impact of memory on answers [31]. However, many patients provided slightly different wording, indicating that even without a wash-out period, they were unable to fully replicate their previous answers. Another limitation is that the follow-up questionnaires were completed at home, resulting in some paper versions being filled out later than the electronic versions. The variations in timing could potentially affect the patients' experiences of their symptoms and, consequently, their responses. Even so, the results showed excellent ICC scores and high agreement in the descriptive data between the electronic and paper versions of the follow-up questionnaires. Regarding loss to follow-up and response rate, four patients never returned the paper versions of the follow-up questionnaires and had to be excluded from that part of the analysis. However, the total response rate, as mentioned above, was very high. In terms of missing data, these were treated differently. For the ICC measurements, missing data in one or both versions were excluded from the analysis, and the same approach was applied to the median difference. For the descriptive data, a missing answer in both the electronic and paper questionnaires was interpreted as agreement. The qualitative analysis may have been influenced by the researchers' preconceptions. To address this, a rigorous and transparent analysis method was employed. Sharing the transcribed interviews with the patients could potentially have uncovered any underlying preconceptions [26]. This equivalence study examined measurement equivalence in a specific patient group, where each patient served as their own control, which limits the generalizability of the findings. Both MYMOP2 and MYCaW have been validated [4,5], as have the Danish versions [20], and MYMOP2 has been used across various settings and patient populations [8], suggesting broad applicability. Furthermore, consistency in setup and design is crucial when changing the administration mode [16]. If these conditions are met, it seems reasonable to assume that similar results could be obtained from other patient groups using a comparable electronic version.

We see great potential in using these questionnaires in electronic form, both because of the possibilities of telemedicine through online and/or phone consultations, and because electronic distribution incurs lower costs [29]. It is also safer to collect and store data electronically in compliance with the General Data Protection Regulation (GDPR) [32]. Since patients define which symptoms or concerns affect them the most, implementing MYMOP2 and/or MYCaW could increase patient involvement and help healthcare professionals prioritize treatment based on the patients’ individual needs and what matters most to them. Additionally, since MYMOP2 and MYCaW also assess well-being, the treatment effect will not only measure changes in symptoms or concerns, as many other PROs do [33,34] but also changes in the patients’ sense of well-being.

## Conclusions

We found a high degree of measurement equivalence between the electronic and paper versions of the MYMOP2 and MYCaW questionnaires, both in terms of intra-rater reliability, median differences in Likert scale responses, and agreement on patient-defined answers within a specific patient group. Semi-structured interviews revealed that most patients preferred the electronic versions of the questionnaires. This preference was primarily due to the convenience and ease of receiving and returning the questionnaires electronically, although some minor disadvantages related to the design of the electronic setup were mentioned. These findings support the use of MYMOP2 and MYCaW in electronic form, which may offer advantages in terms of telemedicine, healthcare efficiency, and patient outcomes.

## Appendices

**Table 4:** Interview guide, translated from Danish.

Introduction	
Purpose	Thank you so much for participating in the validation of the two questionnaires MYMOP2 and MYCaW. We are looking forward to implementing them in the Sequelae Clinic. This interview will last 10-15 minutes, and it will be recorded and transcribed. Both the filled-out questionnaires, the recordings, and the transcriptions will be stored for five years. All data will be handled confidentially, and participants will be anonymized. If you have any questions during the interview, feel free to ask.
Research question	Interview question prompt
Experience of filling out the paper versions of MYMOP and MYCaW	How was it to fill out the paper versions? Can you elaborate …
Experience of filing out the electronic versions of MYMOP and MYCaW	Which electronic devices do you normally use at home? Which device would you use to fill out a questionnaire like these? How was it to fill out the electronic versions? Can you elaborate … Did the digital setup in the electronic questionnaires work? Can you elaborate … Can you give an example … you said, “it worked very well”, why did you think that …
Advantages/disadvantages	How did the two versions differ? How did you experience the difference between answering in hand and digitally? Which version did you prefer and why?
End of the interview	
Sum up and ensure understanding of answers	I will shortly sum up what we have talked about. You said that … is that correct? Now we are about to end the interview, do you have any additions? Thank you again for helping, you are welcome to contact me, if you have any questions.

**Table 5:** Time gaps. Time between completing the electronic and paper follow-up questionnaires for the MYMOP2 and MYCaW (n=26). MYMOP2, Measure Yourself Medical Outcome Profile 2; MYCaW, Measure Yourself Concerns and Wellbeing

Time gap	Patients, n (%)
0 days	14 (54)
1 day	5 (19)
2 days	1 (4)
3 days	1 (4)
5 days	1 (4)
7 days	1 (4)
>10 days	3 (12)
